# Primary Hyperaldosteronism: A Rare Cause of Malignant Hypertension with Thrombotic Microangiopathy in a Kidney Transplant Recipient

**DOI:** 10.1155/2021/9261371

**Published:** 2021-11-15

**Authors:** Carolina Ormonde, Sara Querido, Nuno Rombo, Rita Roque, Belarmino Clemente, André Weigert

**Affiliations:** ^1^Nephrology Department, Hospital do Divino Espírito Santo, Portugal; ^2^Nephrology Department, Centro Hospitalar Lisboa Ocidental-Hospital Santa Cruz, Portugal; ^3^General Surgery Department, Centro Hospitalar Lisboa Ocidental-Hospital Santa Cruz, Portugal

## Abstract

Thrombotic microangiopathy (TMA) is a rare disease that presents with haemolysis and organ damage. The kidney is one of the main affected organs, and TMA is associated with serious complications and increased mortality. In transplanted patients, TMA is even less common and has a variety of possible causes, including thrombotic thrombocytopenic purpura (TTP) and haemolytic-uremic syndrome (HUS), infections, drugs, autoimmune disease, tumours, and malignant hypertension. Transplant-related causes, such as antibody-mediated rejection, calcineurin inhibitors, and viral infections, need to be considered as well. The authors report a rare case of TMA in a kidney transplant recipient, whose investigation revealed malignant hypertension secondary to primary hyperaldosteronism.

## 1. Introduction

Thrombotic microangiopathy (TMA) is a rare disease that presents with arteriolar and capillary thrombosis [1–3]. TMA is a clinicopathologic diagnosis [4]. It can express with a variety of symptoms that reflect haemolysis and organ damage [5]. The kidney is one of the main affected organs [6, 7]. The presence of haemolytic anaemia, thrombocytopenia, and schistocytes in peripheral blood smear establish the diagnosis that can be confirmed by histology of affected organs [4, 5, 8].

Although rare in kidney transplant recipients, with an incidence of 5.6 cases per 1000 renal transplant recipients per year, TMA is a serious complication in these patients. It associates with poor outcomes, both on kidney allograft and patient [3, 9–11].

Thrombotic thrombocytopenic purpura (TTP) and haemolytic-uremic syndrome (HUS) are the two most frequent causes of TMA. Other less common causes are infections (human immunodeficiency virus, cytomegalovirus), drugs (such as chemotherapy agents and ticlopidine), autoimmune diseases (such as systemic lupus erythematosus and scleroderma), disseminated intravascular coagulation, malignant tumours, HELLP syndrome, malignant hypertension, and bone marrow transplant [2, 4–8, 10, 12, 13]. Any of the listed conditions may cause TMA in kidney allograft, but transplant-related causes also need to be considered: antibody-mediated rejection, calcineurin inhibitors, and viral infections (Cytomegalovirus, Epstein-Barr virus, and Polyoma virus) [13, 14].

TMA secondary to malignant hypertension is a very rare entity [15, 16]. The authors report an uncommon case of malignant hypertension secondary TMA in a kidney transplant recipient. To our knowledge, this is the first reported case of TMA secondary to malignant hypertension in a kidney transplant recipient. In addition, in this patients, malignant hypertension was caused by hyperaldosteronism, making it even more unique.

## 2. Case Presentation

A 47-year-old female was evaluated in her routine posttransplant appointment on May 2020. She had a history of end-stage chronic kidney disease due to autosomal dominant polycystic kidney disease. Haemodialysis was initiated in 2005 and she received a deceased donor kidney transplant in 2008. She remained stable for 10 years posttransplant when she developed antibody-mediated acute rejection, for which she was given rituximab and intravenous immunoglobulin, with stabilization but reduced kidney function. Eight months later, she developed a severe CMV colitis with prolonged hospital stay and her baseline creatinine remained at 2.6 mg/dL. Although chronic hypertension had been present for several years, resistant hypertension ensued for about eight months, and she was diagnosed with hypertensive retinopathy two months earlier. She also had hypertensive cardiomyopathy with left ventricular hypertrophy, hypokalaemia with need for potassium supplementation, and previous asthma. Four antihypertensive drugs (carvedilol, nifedipine, clonidine, and enalapril) were insufficient to control adequately blood pressure, and her maintenance immunosuppression included tacrolimus, everolimus, and prednisone.

Her routine lab results revealed a normochromic normocytic anaemia (Hb 8.5 g/dL) with high reticulocyte count (2.9%), no iron or vitamin deficiency and no abnormalities in other blood cell lines. Graft function was stable, and urinalysis revealed mild albuminuria that was already previously present. There were no history of blood loss or associated symptoms. No changes in physical examination were present besides obesity and grade 3 hypertension. She was started on epoetin and reevaluated three weeks later.

On reevaluation, she had worsened anaemia (Hb 6.6 g/dL) and higher reticulocyte count (5.5%). There was also *de novo* thrombocytopenia (109.000/uL), high lactate dehydrogenase (LDH) levels (469 U/L), undetectable haptoglobin, and presence of schistocytes on peripheral blood smear. She was then admitted to the Nephrology Department with the diagnosis of TMA.

**Figure 1:**
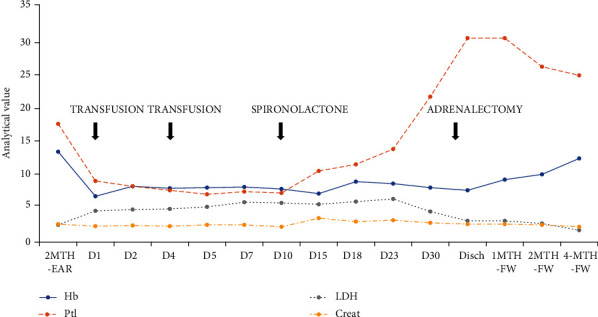
Thrombotic microangiopathy evolution. MTH-EAR: months earlier; Disch: discharge; MTH-FW: months follow-up; Hb: hemoglobin; Ptl: platelets; LDH: lactate desidrogenase; Creat: creatinine. Analytical values presented as hemoglobin in g/dL, platelets in 10^4^/uL, LDH in 10^2^ U/L, and creatinine in mg/dL.

ADAMTS13 and Shiga toxin were negative, and complement activity (C3, C4, AH50, and CH50) was normal; therefore, plasmapheresis was not initiated. Over the first ten days after admission, she presented severe anaemia requiring multiple red blood cell transfusions, platelet downward trend, and high LDH levels (Figure 1). Nevertheless, serum creatinine was stable. All workup for TMA causes was negative, except for HLA class II antibodies that were already previously present and remained at stable levels (Table 1). Kidney graft biopsy was not performed due to high bleeding risk and stable renal function.

**Figure 2:**
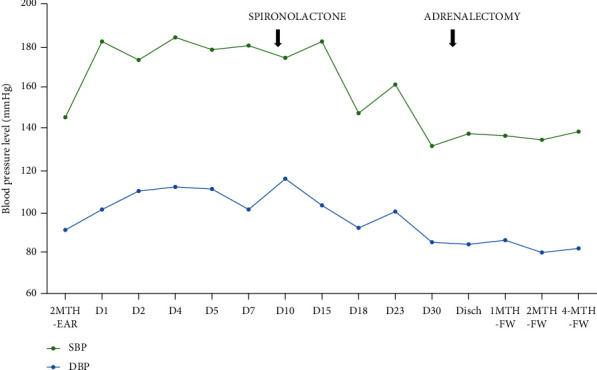
Blood pressure evolution. MTH-EAR: months earlier; Disch: discharge; MTH-FW: months follow-up; SBP: systolic blood pressure; DBP: diastolic blood pressure. Blood pressure values presented as mmHg.

The only abnormal finding on physical examination during hospital-stay, besides excessive weight (body mass index of 34.7 kg/m^2^), was grade 3 hypertension (Figure 2), even under five classes of antihypertensive drugs (previous mentioned plus minoxidil). She also presented persistent hypokalaemia with need for intravenous supplementation. Hypokalaemia workup was suggestive of renal potassium wasting: high urinary potassium excretion (62 meq/L) and a high transtubular potassium gradient (7.0).

**Figure 3:**
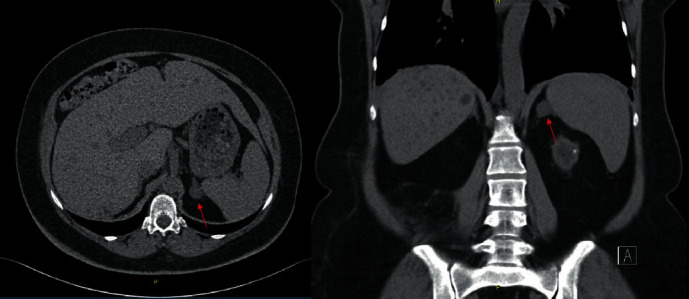
CT scan. Axial and coronal planes of CT scan showing left adrenal mass (indicated by red arrow).

Having excluded almost all TMA causes, we hypothesized that malignant hypertension could be the cause. Secondary hypertension workup was conducted (Table 2), and an adrenal nodule was found in the abdominal CT scan. MRI was suggestive of a cortical adenoma, and blood analysis was compatible with hyperaldosteronism (Figure 3). Pheochromocytoma was excluded. The patient was then started on 50 mg of spironolactone a day. We observed improved control of hypertension (Figure 2) and suspension of potassium supplementation. However, the patient was still under other 5 antihypertensive drugs.

To confirm a unilateral primary hyperaldosteronism, the patient stopped spironolactone to perform an iodo-methyl norcholesterol scintigraphy. However, she developed hypertensive-induced acute pulmonary oedema, and therefore, the exam could not be performed. After stabilization, she was submitted to successful laparoscopic left adrenalectomy. In the postoperative period, she evolved with controlled hypertension under only three antihypertensive drugs (carvedilol, nifedipine, and clonidine) (Figure 2) and no additional need for potassium supplements. At the 13th day after surgery, she was discharged still with hemoglobin level of 7 g/dL, albeit stable and asymptomatic.

On follow-up appointments, she was asymptomatic and with controlled hypertension. Her blood analysis significantly improved. Four months after discharge, she had normal hemoglobin (12.6 g/dL) and LDH levels (208 U/L), normal serum potassium (4.8 meq/L) without need for supplementation, and stable renal function (creatinine 2.4 mg/dL). Chronic vascular changes (left ventricular hypertrophy and hypertensive retinopathy) remained present. Histopathology examination confirmed adrenocortical adenoma.

## 3. Discussion

We report a very unusual case of a kidney transplant patient with TMA secondary to malignant hypertension. After extensive investigation, we concluded that it was caused by primary hyperaldosteronism. TMA is one of the most devastating complications in kidney transplant patients [10]. Regardless of its cause, it begins with and event that triggers endothelial injury. Then, proinflammatory and procoagulant mechanisms are activated and generate haemolysis and platelet aggregation. Microcirculation thrombosis occurs, with consequently organ ischemia. It then becomes a vicious cycle of organ damage [5, 16, 17].

The kidney is one of the most frequent affected organs, along with the nervous system, but any system can be afflicted [5]. TMA's usual clinical findings are haemolytic anaemia, thrombocytopenia, elevated LDH levels, low haptoglobin level, and schistocytes in the peripheral blood smear [12, 14].

Apart from the common TMA causes, such as TTP, HUS, drugs, malignant tumours, and auto-immune diseases, in renal transplant patients, other specific causes need to be considered: antibody-mediated acute rejection, calcineurin inhibitors, and viral infections [3, 10, 13, 18].

Malignant hypertension is a rare cause of TMA. Its incidence is around 1% of all hypertensive patients [15, 16]. Retinopathy is the most frequent clinical sign, and some patients may have papilledema [19, 20]. Acute kidney injury, acute heart failure, and hypertensive encephalopathy might also be present [17]. Laboratorial changes are similar to other causes of TMA [19]. Support measures and hypertension control are the pillars of treatment, although sometimes plasmapheresis is initiated until laboratory results exclude HUS or TTP. If a secondary cause for hypertension is unveiled, it must be specifically treated as well [5, 15, 20–23]. A study by Cavero et al. suggested that eculizumab could be beneficial in malignant hypertensive TMA, irrespective of complement abnormalities [20].

After ruling out most of TMA common causes, we hypothesized that our patient's uncontrolled hypertension could explain this clinical condition. In the setting of a 47-year-old woman with new onset resistant hypertension with end organ damage (recently diagnosed severe hypertensive retinopathy and cardiomyopathy), we studied potential secondary causes. Her unexplained long-term hypokalaemia with high transtubular potassium gradient led us to suspect of hyperaldosteronism, which was then confirmed by the extended study. Pheochromocytoma was excluded by catecholamine analysis and MIBG scintigraphy. She was then diagnosed with a primary hyperaldosteronism due to a functional adrenal adenoma. Although not confirmed by iodo-methyl norcholesterol scintigraphy, impossible to perform due to life-threatening risks of the interruption of spironolactone, all other studies were suggestive, along with the hypertension and hypokalaemia improvement with spironolactone. The fact that she developed acute hypertensive pulmonary oedema after suspending this drug was also very suggestive. The diagnosis was then confirmed by histology of the adrenal mass. Also, gradual improvement of acute laboratory and clinical features in the following weeks and months corroborated our suspicion. Chronic vascular changes remained as it would be expected due to previous long-standing hypertension.

Antibody-mediated acute rejection was the only cause of TMA that was not completely excluded throughout our investigation. To exclude this hypothesis, we would need to perform a graft biopsy, difficult to justify both because of the major risk of bleeding associated with thrombocytopenia, anaemia, and severe hypertension, and because her renal function and proteinuria remained stable during these events, making rejections a less likely explanation. Lastly, even if she had an antibody-mediated rejection, we would be reluctant to give additional immunosuppression due to her previous significant infectious complications.

There are several TMA cases due to malignant hypertension reported in the literature but few in kidney transplant patients or secondary to hyperaldosteronism. Basturk and Pamukcu reported a case of TMA caused by malignant hypertension due to hyperaldosteronism that was successfully treated with spironolactone [23]. In this particular case, an adrenal nodule was not identified. There are recent studies that report that high aldosterone levels could have an important role on malignant hypertension TMA's pathophysiology [24]. Akimoto et al. showed a positive correlation between aldosterone and LDH levels in hypertensive patients either with or without hyperaldosteronism [19].

In kidney transplanted patients, the differential diagnosis of TMA is wide, and all causes of *de novo* or recurrent TMA in the graft must be investigated. We report a very rare case of malignant hypertension TMA due to primary hyperaldosteronism in a kidney transplant patient.

## Figures and Tables

**Table 1:** Thrombotic microangiopathy workup.

Workup	Result (normal range)
INR	1.0 (0.8-1.1)
aPTT	33.8 seconds (23-38)
Fibrinogen	5.66 g/L (1.5-4.0)
ADAMTS13 activity	67% (50-160)
Shiga toxin	Negative
C3	136 mg/dL (80-178)
C4	31.6 mg/dL (12-42)
Functional complement studies	Normal
Genetic complement studies	Normal
Antinuclear antibodies	Negative
Anti-dsDNA antibodies	Negative
Antiphospholipid antibodies	Negative
Extractable nuclear antigen antibodies	Negative
Serum protein electrophoresis	Normal
Vitamin B12	234 pmol/L (141-489)
Homocysteine	13.3 *μ*gmol/L (<15)
Serum CMV viral load	Negative
Serum and urinary BK and JC viral load	Negative
Serum parvovirus B19 viral load	Negative
Anti-HIV 1 and 2 antibodies	Negative
Anti-HCV antibodies	Negative
HBs antigen and anti-HBc antibodies	Negative
Class I HLA antibodies	Negative
Class II HLA antibodies	Positive for 5 specificities, nondonor specific, maximum 15.600 MFI

**Table 2:** Secondary hypertension workup.

Workup	Result (normal range)
24-hour urinary cortisol	25.5 *μ*g/24 h (4.3-45)
Plasma renin activity (PRA)	4.6 ng/mL/h (06-4.3)
Serum aldosterone	144.6 ng/dL (4.0-31.0)
Aldosterone/PRA	31 (<30)
24-hour urinary metanephrines	
Normetanephrine	284 *μ*g (<632)
Metanephrine	104 *μ*g (<276)
3-Methoxythyramine	144 *μ*g (<426)
TSH	4.68 *μ*UI/mL (0.5-5.0)
Free T4	15.8 pmol/L ng/dL (12-30)
Renal Doppler	Without renal artery stenosis
Abdominal CT and MRI	Left adrenal nodule 2 × 1.5 cm
MIBG scintigraphy	Negative for neural crest tumours
